# Behavioral and Molecular Consequences of Pathogenic Tau Expression in Mouse Locus Coeruleus

**DOI:** 10.1002/alz.090727

**Published:** 2025-01-03

**Authors:** Anuradha Korukonda, Jake Atallah, Margaret M Tish, David Weinshenker

**Affiliations:** ^1^ Emory University School of Medicine, Atlanta, GA USA

## Abstract

**Background:**

Early stages of Alzheimer’s disease (AD) are characterized by neuropsychiatric symptoms such as anxiety, apathy, compulsivity, and sleep disturbances, which manifest years before cognitive deficits. It has been hypothesized that dysregulation of the locus coeruleus‐norepinephrine (LC‐NE) system contributes to these symptoms because (1) the LC is the first site where hyperphosphorylated ‘pretangle’ tau can be detected in the human brain and (2) NE influences physiological processes such as mood, stress responses, and arousal. To investigate causal relationships between LC tau pathology and neuropsychiatric symptoms, we developed a translationally‐relevant model where pathogenic tau is exclusively expressed in mouse LC to recapitulate the ‘LC‐first’ phenomenon.

**Method:**

Adult male and female tyrosine hydroxylase (TH)‐Cre mice aged 2‐3 months received intra‐LC infusions with a Cre‐dependent adeno‐associated virus expressing either EYFP or P301S mutant human tau (n = 10/group). Three months following infusions, mice were tested in a panel of behaviors encompassing neuropsychiatric and cognitive domains. Following behavioral testing, brain sections containing the LC and projection regions such as dentate gyrus and prefrontal cortex were immunostained for human tau and phospho‐tau (AT8) to verify hyperphosphorylated tau expression, TH to evaluate LC integrity, NE transporter (NET) to assess NE innervation, and the neuroinflammatory markers GFAP and Iba‐1.

**Result:**

P301S human tau‐expressing mice displayed significantly lower latency to fall asleep after gentle handling, indicating hypersomnia. Additionally, they showed increased latency to eat a food pellet in a novel arena, reflecting anxiety‐like behavior, and stress‐induced compulsivity in nestlet shredding compared to EYFP‐expressing mice. In the fear conditioning paradigm, mice associated tone with foot shock across both treatment groups during training, and exhibited normal context and cued memory of the shocks as assessed by time spent freezing. AT8 staining indicated hyperphosphorylated tau accumulation in cell bodies and somatodendritic compartments of TH+ neurons. However, tau pathology did not affect LC cell body integrity (TH and NET immunoreactivity) but triggered astrocytic (GFAP) and microglial (Iba‐1) reactivity compared to EYFP controls.

**Conclusion:**

At 3 months, anxiety‐ and compulsive‐like behaviors and hypersomnia phenotypes suggest dysregulation of LC‐NE transmission. Further studies are ongoing to assess the cellular and molecular mechanisms governing these behavioral symptoms in early AD.

